# Network Analysis of the Potential Role of DNA Methylation in the Relationship between Plasma Carotenoids and Lipid Profile

**DOI:** 10.3390/nu11061265

**Published:** 2019-06-04

**Authors:** Bénédicte L. Tremblay, Frédéric Guénard, Benoît Lamarche, Louis Pérusse, Marie-Claude Vohl

**Affiliations:** 1 Institute of Nutrition and Functional Foods (INAF), Laval University, 2440 Hochelaga Blvd, Quebec City, QC G1V 0A6, Canada; benedicte-l.tremblay.1@ulaval.ca (B.L.T.); frederic.guenard@fsaa.ulaval.ca (F.G.); benoit.lamarche@fsaa.ulaval.ca (B.L.); louis.perusse@kin.ulaval.ca (L.P.); 2 School of Nutrition, Laval University, 2425 rue de l’Agriculture, Quebec City, QC G1V 0A6, Canada; 3 Department of Kinesiology, Laval University, 2300 rue de la Terrasse, Quebec City, QC G1V 0A6, Canada

**Keywords:** carotenoids, DNA methylation, french canadians, hub genes, lipid profile, WGCNA

## Abstract

Variability in plasma carotenoids may be attributable to several factors including genetic variants and lipid profile. Until now, the impact of DNA methylation on this variability has not been widely studied. Weighted gene correlation network analysis (WGCNA) is a systems biology method used for finding gene clusters (modules) with highly correlated methylation levels and for relating them to phenotypic traits. The objective of the present study was to examine the role of DNA methylation in the relationship between plasma total carotenoid concentrations and lipid profile using WGCNA in 48 healthy subjects. Genome-wide DNA methylation levels of 20,687 out of 472,245 CpG sites in blood leukocytes were associated with total carotenoid concentrations. Using WGCNA, nine co-methylation modules were identified. A total of 2734 hub genes (17 unique top hub genes) were potentially related to lipid profile. This study provides evidence for the potential implications of gene co-methylation in the relationship between plasma carotenoids and lipid profile. Further studies and validation of the hub genes are needed.

## 1. Introduction

Cardiometabolic (CM) diseases comprise conditions ranging from insulin resistance and metabolic syndrome to cardiovascular disease and type-2 diabetes [1]. Healthy eating, including the consumption of fruits and vegetables, is associated with a favorable CM health [2]. Carotenoids, which are reliable biomarkers of fruit and vegetable intakes, are composed of hundreds of fat-soluble pigments [3]. However, six main carotenoids (α-carotene, β-carotene, β-cryptoxanthin, lutein, lycopene, and zeaxanthin) represent over 95% of total circulating carotenoids in human [4,5]. Variability among individuals in circulating carotenoids may be due to several factors, including age, sex, body weight, genetics, and lipid profile [3]. Lower circulating carotenoid concentrations are associated with lower plasma total cholesterol (TC), LDL-cholesterol (LDL-C), and HDL-cholesterol (HDL-C) concentrations [6]. Moreover, α- and β-carotene correlated with HDL-C and triglyceride (TG) concentrations [7], while β-crytoxanthin and zeaxanthin correlated with TG concentrations in a previous study by our group [8]. Thus, several studies have observed associations between circulating carotenoids and plasma lipid concentrations, which are both transported by lipoproteins [9].

Genetic variants have been shown to influence circulating carotenoid concentrations by causing differences in the absorption, assimilation, distribution, metabolism, and excretion of carotenoids [10,11,12,13,14,15]. Carotenoids and their derivatives (e.g., retinoid) have also been shown to modulate gene expression via several transcriptional systems [16]. However, very few studies have documented the relationship between carotenoids and DNA methylation, at least from a genome-wide viewpoint. In cell studies, lycopene had modest to null effects on DNA methylation of *GSTP1*, which is involved in prostate and breast cancers [17,18]. Moreover, a study of 165 overweight and obese subjects revealed an association between DNA methylation of *HERV*-w and *TNFα* in blood leukocytes and dietary intakes of β-carotene and carotenoids [19]. Interestingly, the reported protective effects of high fruit and vegetable intake on age-related diseases (coronary heart disease, stroke, type-2 diabetes) may be mediated by the association between blood carotenoids levels and extrinsic epigenetic age acceleration, which is a measure of epigenetic age [20]. Thus, circulating carotenoids and dietary intakes of carotenoids seem to impact DNA methylation. Moreover, there is more and more evidence that DNA methylation plays a role in the regulation of blood lipid levels and lipid metabolism-linked phenotypes and diseases [21]. No previous study has considered the involvement of genome-wide DNA methylation levels in the association between plasma carotenoids and lipid profile. Weighted gene correlation network analysis (WGCNA) is a widely used systems biology approach designed for high dimensional data (i.e. gene expression, DNA methylation, metabolites etc.) [22]. It allows finding gene clusters (modules) with highly correlated DNA methylation levels, relating these modules to phenotypic traits, and identifying key hub genes within modules that are related to phenotypic traits [22].

The objective of the present study was to examine the role of DNA methylation in the relationship between plasma carotenoid concentrations and lipid profile using WGCNA in 48 healthy subjects. First, cytosine-phosphate-guanine (CpG) sites whose DNA methylation levels are associated with carotenoid concentrations were identified using linear regressions. Second, WGCNA was used to identify specific modules and key hub genes related to lipid profile traits. The hypotheses were that genome-wide DNA methylation levels in blood leukocytes are associated with plasma total carotenoid concentrations and that clusters of genes associated with carotenoids are also correlated to lipid profile traits. Our results highlighted the potential implication of gene co-methylation in the relationship between plasma carotenoids and lipid profile.

## 2. Materials and Methods

### 2.1. Patients and Design

The GENERATION Study aimed at evaluating familial resemblances in omics (DNA methylation [23] and gene expression [24]) and metabolic (metabolites [25] and carotenoids [8]) profiles in healthy families. The GENERATION Study comprises a total of 48 French-Canadian subjects from 16 families of Quebec City (Canada). Families composed of 16 mothers, six fathers, and 26 children lived under the same roof. Families comprised at least the mother and one child (8–18 years old). Parents had to be the biological parents of their child (or children), non-smokers, with body mass index (BMI) ranging between 18 and 35 kg/m^2^, and free of any metabolic conditions requiring treatment (Synthroid^®^ (levothyroxine) and oral contraceptive were tolerated). Children also had to be non-smokers, in good general health and not using psycho-stimulators (Ritalin^®^ (methylphenidate), Concerta^®^ (methylphenidate), and Strattera^®^ (atomoxetine)). Blood samples were taken from both parents and children at the Institute of Nutrition and Functional Foods (INAF). The experimental protocol was approved by the Ethics Committees of Laval University Hospital Research Center and Laval University. All participants signed an informed consent document. Parental consent was also obtained by signing the child consent document.

### 2.2. Anthropometric and CM Measurements

Body weight and height were measured [26]. Blood samples were collected from an antecubital vein into vacutainer tubes containing EDTA after 12-hour fast and 48-hour alcohol abstinence. Plasma was separated by centrifugation (2500 g for 10 min at 4 °C), and samples were aliquoted and frozen (−80 °C). Plasma TC and TG concentrations were measured using enzymatic assays [27,28]. Precipitation of very-low density lipoprotein and LDL particles in the infranatant with heparin manganese chloride generated the HDL-C fraction for measurements of HDL levels [29]. LDL-C was estimated using the Friedewald formula [30]. The rocket immunoelectrophoretic method was used to measure plasma apolipoprotein B-100 (ApoB100) concentrations [31]. Plasma C-reactive protein (CRP) was measured by nephelometry using a sensitive assay (Prospec equipment Behring) [32]. Fasting plasma glucose concentrations were enzymatically measured [33]. Radioimmunoassay with polyethylene glycol separation was used to measure fasting plasma insulin concentrations [34].

### 2.3. DNA Extraction and Methylation Analysis

Genomic DNA was extracted from blood leukocytes using the GenElute Blood Genomic DNA kit (Sigma-Aldrich, St. Louis, MO, USA) in all 48 subjects. NanoDrop Spectrophotometer (Thermo Scientific, Wilmington, DE, USA) and PicoGreen DNA methods were used to quantify DNA. Infinium Human Methylation 450 array (Illumina, San Diego, CA, USA) was used to measure DNA methylation levels. McGill University and Genome Quebec Innovation Center (Montreal, QC, Canada) proceeded to the bisulfite conversion and quantitative DNA methylation analysis. Methylation data on all 485,577 CpG sites were analyzed using Illumina GenomeStudio software v2011.1 and the Methylation Module. All samples were retained after quality control steps [23]. GenomeStudio was used to perform global normalization using control probes. Probes with a detection *p*-value > 0.01 in more than 5 subjects (>10% of all subjects) were removed. We restricted our analysis to autosomes and multi-mapped probes were also excluded [35]. Thus, 472,245 probes were considered in the analysis.

### 2.4. Carotenoid Measurements

Samples and standards preparation has been reported previously [8]. Briefly, 100 µL of fasting plasma samples were thawed a few hours before analysis and transferred to Eppendorf tubes with 20 µL of 2-propanol, and 20 µL of carotenoid standard. Samples were transferred on a 400 μL fixed well plate (ISOLUTE^®^ SLE+, Biotage, Charlotte, NC, USA) with 900 µL of hexane:isopropanol (90/10, *v*/*v*) in each well. Each extracted sample was evaporated under nitrogen and reconstituted with 300 µL of methanol:dichloromethane (65/35, *v*/*v*). Plates were shaken for 10 min and samples were transferred into high performance liquid chromatography glass vials.

Agilent 1260 liquid handling system (Agilent, Mississauga, ON, Canada) was used to perform high performance liquid chromatography (HPLC)-UV analysis as previously described [8]. Carotenoids were separated with a mobile phase consisting of methanol:water (98/2, *v*/*v*; Eluent A) and methyl-tert-butyl ether (MTBE; Eluent B; VWR, Mississauga, ON, Canada). Flow-rate and gradient elution are detailed in a previous paper [8]. UV detector was set at 450 nm and identification of each compound was confirmed using retention time and UV spectra (190–640nm) of the pure compounds. The Chemstation software (Agilent, Mississauga, ON, Canada) was used to carry out data acquisition. The carotenoid concentrations are reported in μmol/L of plasma. One value for β-crypoxanthin was considered an outlier (defined as a value falling outside of the mean ± 4 standard deviations) and was excluded from analyses. The exclusion of this outlier is likely to have very little impact on further analysis, considering that it affected only one subject, and the mean of total carotenoids remained very similar (6.00 μmol/L without the outlier and 6.04 μmol/L including the outlier).

### 2.5. Association between DNA Methylation Levels and Total Carotenoid Concentrations

Concentrations of plasma carotenoids are available in Supplementary Table S1. Plasma total carotenoid concentrations (µmol/L of plasma) were defined as the sum of α-carotene, β-carotene, β-cryptoxanthin, lutein, lycopene, and zeaxanthin concentrations. Regressions between normalized DNA methylation levels of all 472,245 CpG sites and total carotenoid concentrations adjusted for the family ID were computed using R software v2.14.1 (R Foundation for Statistical Computing; http://www.r-project.org) [36]. Plasma total carotenoid concentrations (independent variable) were used to predict DNA methylation levels (dependent variable). A *p*-value ≤ 0.05 was used to identify significant associations. Regressions were adjusted for family ID (fixed effect) to account for the familial structure. In order to maintain a more exploratory approach, the regressions were not adjusted for other confounding factors. Choices of linear model and confounding factors were made for comparison purposes with similar study by our group [37]. Weighted gene correlation network analysis (WGCNA) was performed with methylation levels of 20,687 CpG sites showing a significant association (*p*-value ≤ 0.05) with total carotenoid concentrations. This allowed evaluating co-methylation similarities only in CpG sites that are associated with carotenoids.

### 2.6. Weighted Gene Correlation Network Analysis (WGCNA)

WGCNA was performed with the WGCNA package [22,38] in R software using default parameters [36]. A weighted adjacency matrix was established by calculating Pearson correlations between each CpG site pair. The co-methylation similarity matrix was raised to a power β = 5 (R^2^ of 0.9570) to calculate weighted adjacency matrix [39] (Supplementary Figure S1). From this matrix, values were used to construct a topological overlap matrix (TOM), which provided a similarity measure. The TOM is then used to calculate the corresponding dissimilarity (1-TOM). Next, CpG sites with coherent methylation profiles were grouped into modules using the average linkage hierarchical clustering applied to the TOM-based dissimilarity [39]. The dynamic tree cutting algorithm (function = cutreeDynamic, deep split = 0, minimum number of genes per module = 40, cut height = 0.25) was used to detect methylation modules (clusters of densely interconnected CpG sites in terms of co-methylation). The assignment of outlying genes to modules was performed using the Partitioning Around Medoids (PAM) method. DNA methylation levels of CpG sites within a module were summarized with the module eigengene (ME) value, which is the overall methylation level of CpG sites clustering in a module. To identify modules that were significantly associated with lipid profile traits (TC, LDL-C, HDL-C, TG, ApoB100), correlation between MEs [40] and traits were computed. Associations of individual genes with lipid profile traits was quantified using gene significance (GS), defined as the absolute correlation between the gene and the trait. Module membership (MM), defined as the correlation of the ME and the gene methylation profile, was used to quantify the similarity of all genes to each module. Correlations between MM and GS were computed to identify modules of interest. We considered CpG sites with the highest MM and GS to be those of highest biological relevance [41]. CpG sites with a GS > 0.2, a MM > 0.8, and a *p*-value ≤ 0.05 were retained as hub genes. Due to the high number of hub genes obtained (*n* = 2734), genes were subsequently ordered according to their GS with the trait. A total of three top annotated hub genes were selected in each of the six module-lipid associations, for a total of 18 top hub genes. VisANT 5.0 was used to construct and visualize the topological interaction network in the black, turquoise, and pink modules [42]. VisANT is a software framework for mining, analyzing and visualizing hierarchical organization of biological networks [43]. Weighted correlation cut-offs of 0.23, 0.44, and 0.27 were used for the black, turquoise, and pink modules, respectively. The cut-offs were chosen to obtain visually interpretable networks, without being too heavily loaded. Central genes were those with the most gene-gene connections. Enrichr (http://amp.pharm.mssm.edu/Enrichr/) was used to obtain KEGG pathways 2019 and Gene Ontology molecular functions 2018 associated with genes in the black, turquoise, and pink modules [44].

## 3. Results

### 3.1. Characteristics and Biochemical Parameters

Characteristics and biochemical parameters including plasma total carotenoid concentrations of the parents and children are presented in Table 1. Plasma concentrations of the six main carotenoids (α-carotene, β-carotene, β-cryptoxanthin, lutein, lycopene, and zeaxanthin) and of total carotenoids concentrations measured in each participant are presented in Supplementary Table S1.

### 3.2. Weighted Gene Correlation Network Analysis (WGCNA)

Normalized methylation levels of all 472,245 CpG sites were tested for associations with plasma total carotenoid concentrations using regressions. A total of 20,687 CpG sites were significantly associated (*p* ≤ 0.05) with total carotenoids (Supplementary Table S2). This subset of CpG sites associated with total carotenoids was then related to lipid profile traits using WGCNA.

**Figure 1:**
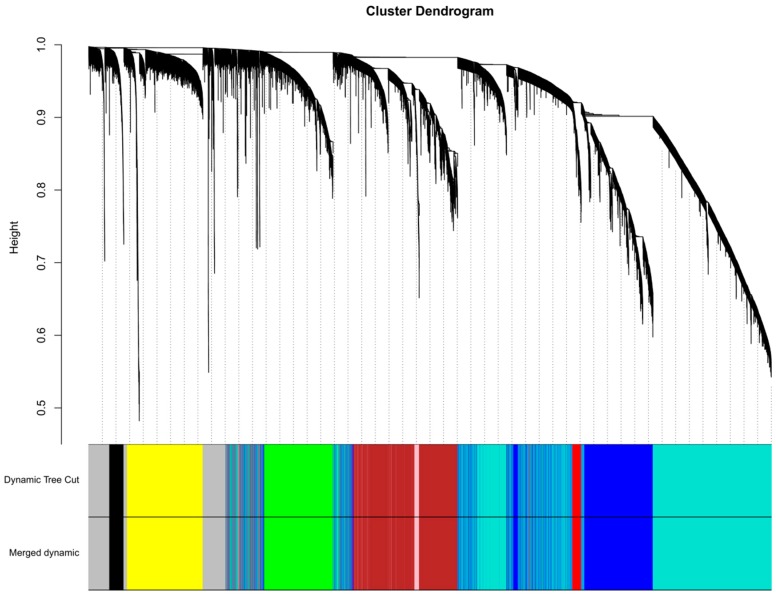
Gene dendogram obtained using average linkage hierarchical clustering. The dynamic tree cut yielded nine modules. The merged dynamic yielded same modules as the dynamic tree cut using a cut-off of 0.25. Module colors are shown correspondingly.

**Figure 2:**
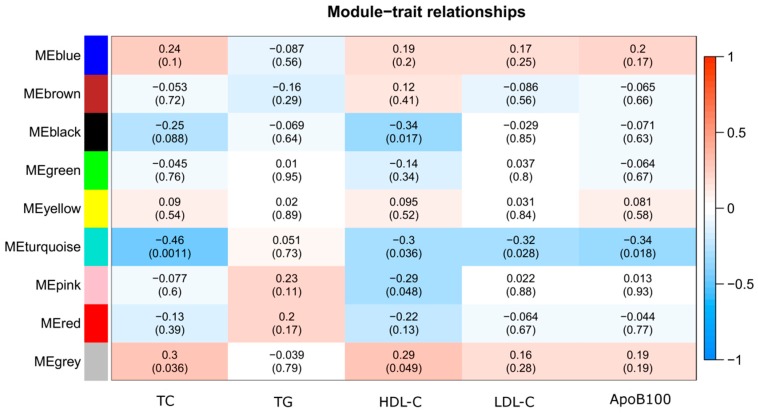
Heatmap of module-trait relationships depicting correlations between module eigengenes and lipid profile traits. Numbers in the table correspond to the correlation *r* and the *p*-value in parentheses. The degree of correlation is illustrated with the color legend. Abbreviations: Apolipoprotein B100 (ApoB100), high-density lipoprotein cholesterol (HDL-C), low-density lipoprotein cholesterol (LDL-C), module eigengene (ME), total cholesterol (TC), triglycerides (TG).

A total of nine distinct modules were identified from methylation levels of the 20,687 CpG sites associated with total carotenoid concentrations using a dynamic tree cutting algorithm (Figure 1). Using a cut-off height of 0.25 for the clustering of module eigengenes (MEs) (merged dynamic), none of the modules has merged (Figure 1). The black, blue, brown, green, grey, pink, red, turquoise, and yellow modules contained 431, 3308, 3235, 2096, 1479, 243, 482, 7119, and 2294 CpG sites, respectively. The 1479 uncorrelated CpG sites were assigned to the grey module, which was excluded from further analysis. Correlations between MEs and lipid profile traits (TC, LDL-C, HDL-C, TG, ApoB100) were computed to find modules of interest. According to the heatmap of module-trait correlations (Figure 2), ME of the black module (431 CpG sites) had a negative correlation with HDL-C (*r* = −0.34 *p* = 0.017). The ME of the turquoise module (7119 CpG sites) had negative correlations with TC (*r* = −0.46 *p* = 0.0011), HDL-C (*r* = −0.30 *p* = 0.036), LDL-C (*r* = −0.32 *p* = 0.028), and ApoB100 (*r* = −0.34 *p* = 0.018). The ME of the pink module (243 CpG sites) had a negative correlation with HDL-C (*r* = −0.29 *p* = 0.048).

Gene significance (GS) (i.e., correlation between CpG site methylation and lipid profile traits) was correlated to module membership (MM) (i.e., correlation of the ME and the CpG site methylation profile). In the black module, GS for HDL-C had a significant correlation with the MM (*r* = 0.25 *p* = 1.5 × 10^−7^) (Supplementary Figure S2). In the turquoise module, GS for TC, HDL-C, LDL-C, and ApoB100 had significant correlations with the MM (*r* = 0.46 *p* < 1 × 10^−200^, *r* = 0.17 *p* = 2.6 × 10^−47^, *r* = 0.41 *p* < 1 × 10^−200^, and *r* = 0.43 *p* < 1 × 10^−200^, respectively) (Supplementary Figure S2). In the pink module, GS for HDL-C had a significant correlation with the MM (*r* = 0.37 *p* = 2.7 × 10^−9^) (Supplementary Figure S2). Hence, the black, turquoise, and pink modules were considered as modules of interest for subsequent analyses.

### 3.3. Hub Gene Analysis

Hub gene analysis in black, turquoise, and pink modules was conducted to explore the underlying mechanisms behind the associations observed. A total of 17, 2700, and 17 hub genes were identified in the black, turquoise, and pink modules, respectively (Supplementary Table S3). Due to their high number, hub genes were subsequently ordered according to their GS with the trait. Top three most significant hub genes were selected in each module-lipid association, for a total of 18 top hub genes (Table 2). In the black module, *PAX4*, *TBC1D16*, and *PGA5* were the top three hub genes associated with HDL-C. In the turquoise module, *RBL2*, *GRIN3A*, and *TEX2* were the top three hub genes associated with TC, while *LST1*, *GOSR1*, and *GBGT1* were associated with HDL-C, *C7orf50*, *PDPK1*, *SYT17* with LDL-C, and finally *GABBR1*, *RAG2*, and *C7orf50* were associated with ApoB100 (Table 2). In the pink module, *RNASE11*, *TRIM68*, and *DEPDC1* were the top three hub genes associated with HDL-C (Table 2).

### 3.4. Topological Interaction Networks

**Figure 3:**
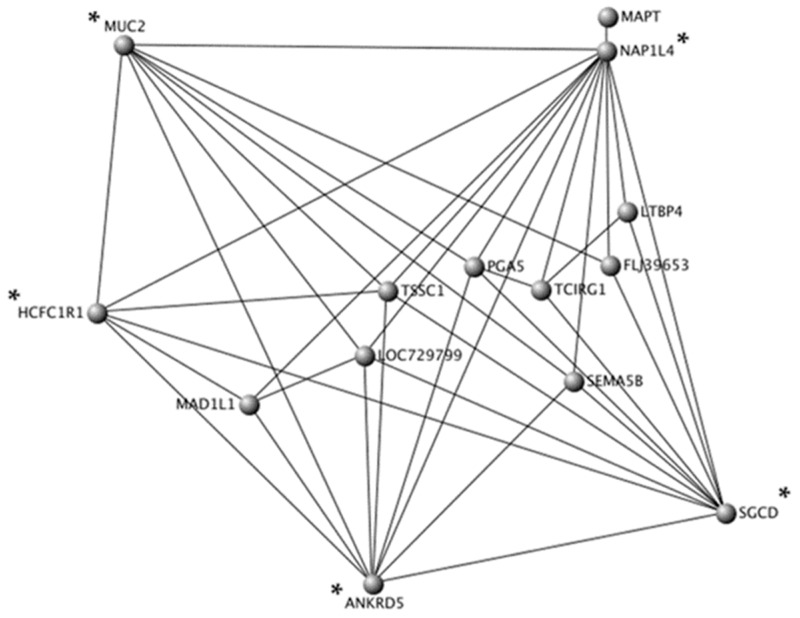
Topological interaction network of 14 unique genes in the black module. Each gene is represented by a node and edge number is proportional to connection strength. Gene-gene interaction network was constructed and visualized using VisANT 5.0. Central genes are identified using asterisks.

**Figure 4:**
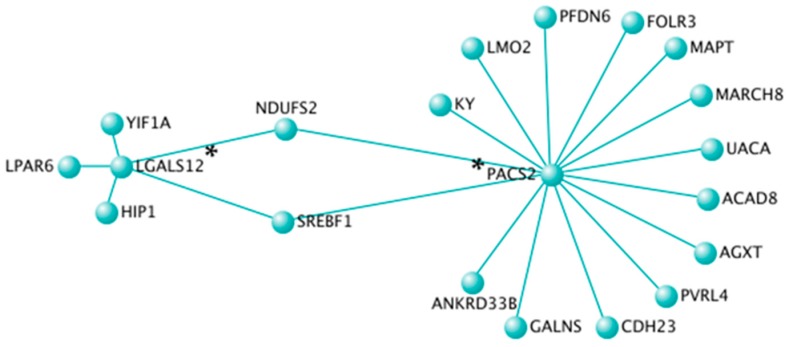
Topological interaction network of 20 unique genes in the turquoise module. Each gene is represented by a node and edge number is proportional to connection strength. Gene-gene interaction network was constructed and visualized using VisANT 5.0. Central genes are identified using asterisks.

**Figure 5:**
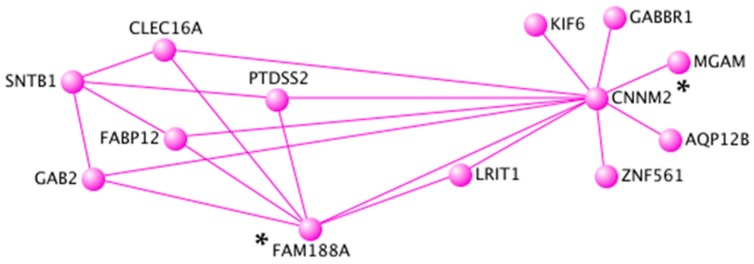
Topological interaction network of 13 unique genes in the pink module. Each gene is represented by a node and edge number is proportional to connection strength. Gene-gene interaction network was constructed and visualized using VisANT 5.0. Central genes are identified using asterisks.

Topological interaction networks were used to illustrate gene-gene interactions within the black, turquoise, and pink modules. In order not to overload networks, weighted correlation cut-offs were used. In the black module containing 431 CpG sites, only annotated CpG sites with a weighted correlation of 0.23 were included (*n* = 14 unique genes) (Figure 3). In the turquoise module containing 7119 CpG sites, only annotated CpG sites with a weighted correlation of 0.44 were included (*n* = 20 unique genes) (Figure 4). In the pink module containing 243 CpG sites, only annotated CpG sites with a weighted correlation of 0.27 were included (*n* = 13 unique genes) (Figure 5). Gene Ontology molecular functions and KEGG pathways of genes in the black, turquoise and pink modules are presented in Supplementary Table S4.

## 4. Discussion

First, in order to identify CpG sites associated with carotenoids, associations between genome-wide DNA methylation levels and plasma total carotenoid concentrations were tested. A total of 20,687 CpG sites were significantly associated with total carotenoid concentrations. To the best of our knowledge, this is the first study that considered the association of plasma total carotenoid concentrations with genome-wide DNA methylation levels. Until now, studies have been mainly focusing on the effect of genetic variants (SNPs) on the variability in carotenoid concentrations [45,46,47]. Circulating carotenoids have also been shown to have an impact on gene expression via transcription factors [16]. However, very few studies have considered the impact of carotenoids on DNA methylation. In two cell studies, lycopene had modest to null effects on DNA methylation of *GSTP1*, which is involved in prostate and breast cancers [17,18]. In addition, a study of 165 overweight and obese subjects observed positive associations between *HERV*-w methylation and dietary intakes of β-carotene and carotenoids, while *TNFα* methylation showed negative associations with dietary intakes of β-carotene and carotenoids [19]. Moreover, blood carotenoid levels were also associated with extrinsic epigenetic age acceleration [20]. Thus, the relationship between carotenoids and DNA methylation has not been widely studied using pan-genomic tools. A potential mechanism underlying the action of carotenoids on DNA methylation is the inhibition of the DNA methyltransferase (DNMT). Studies have shown that carotenoids (lycopene and astaxanthin) decreased DNMT activity and protein level in vitro [48,49].

Secondly, modules and key hub genes related to lipid profile traits in the subset of 20,687 CpG sites associated with carotenoid concentrations were identified using WGCNA. Among nine modules identified, MEs of the black and pink modules had a significant correlation with HDL-C, while ME of the turquoise module had significant correlations with TC, HDL-C, LDL-C, and ApoB100. Thus, highly co-methylated genes in the black, turquoise, and pink modules may have potential interactions or biological effects on lipid concentrations. Moreover, these modules also showed positive correlations between GS for several lipid profile traits and the MM of the respective module. This suggests that genes showing a strong association with lipid profile traits were also the most important elements of their respective module. The analysis with hub genes allowed getting insights on the potential mechanisms linking carotenoid concentrations and lipid profile. Three top hub genes were selected in each of the six module-lipid associations. Among these 18 top hub genes (17 unique top hub genes), several were related to lipid profile. First, cg07665923 is located in *C7orf50*. Several SNPs within this gene have been associated with TC. Indeed, SNP rs1997243 was associated with TC in a genome-wide association study on 188,577 individuals [50]. Moreover, another SNP (rs6951245) has been shown to be a novel pleiotropic locus for both CRP and TC [51]. SNP rs11763835 is also a peak cis-microRNA-eQTL associated with TC [52]. Second, cg02500883 is located in *GRIN3A*, encoding for a glutamate ionotropic receptor NMDA type subunit 3A. SNPs within this gene have been associated with circulating lipid concentrations [53,54]. Interestingly, treatment with astaxanthin, a xanthophyll carotenoid, in rat neurons reduced *GRIN3A* gene expression [55]. Third, cg21671607 is located in *RAG2*, encoding for the recombination activating 2. Variations in this gene are associated with several clinical phenotypes going from severe, early-onset infections to inflammation and autoimmunity [56]. Interestingly *ApoE* and *RAG2*-deficent mice have lower plasma TC levels than do immune-competent apoE mice suggesting an effect of the immune system on plasma lipid homeostasis [57]. Finally, cg24240870 is located in *GOSR1* (*GS28*), encoding for the Golgi SNAP receptor complex member 1, which transports proteins among the endoplasmic reticulum and the Golgi, and between Golgi compartments. In murine melanocytes, 25-hydroxycholesterol reduced protein levels of GS28 and may be linked to cholesterol homeostasis through an OSBP-related protein [58].

Moreover, topological interaction network visualization represented another method to identify novel central genes in co-methylation modules. In the black module, five central genes were identified (*ANKRD5*, *HCFC1R1*, *MUC2*, *NAP1L4*, and *SGCD*). A genetic variant (rs7935422) in *NAP1L4*, encoding for a nucleosome assembly protein 1 like 4, has been associated with HDL-C concentrations in various populations [59]. Moreover, genetic variants in *SGCD*, encoding for a sarcoglycan delta, have been associated with circulating lipid concentrations [54] and HDL particle size [60]. In the turquoise module, two central genes were identified (*PACS2* and *LGALS12*). Interestingly, *PACS2* impacts lipid metabolism by controlling formation of endoplasmic reticulum lipid-synthesizing centers, which are found on mitochondria-associated membranes [61]. Moreover, *LGALS12* is encoding for the galectin 12. Expression of *LGALS12* in adipocytes is up-regulated by PPAR-γ agonists suggesting its role in insulin signaling and type-2 diabetes [62]. In the pink module, two central genes were identified (*CNNM2* and *FAM188A*). A genetic variant in *CNNM2*, encoding for a cyclin and CBS domain divalent metal cation transport mediator 2, has been associated with coronary artery disease and blood pressure [63,64,65]. In summary, several hub genes and central genes were related to lipid metabolism, immune and inflammatory response, and the sensible connection between the two.

The present study has some limitations. The main limitation lies in the small sample size, which may reduce variability in DNA methylation levels, as well as limiting the statistical power to detect CpG sites associated with total carotenoid concentrations. We did not account for multiple testing, since we used an exploratory approach and did not want this study to be overly constrained. However, WGCNA allowed reducing the impact of multiple testing by grouping the 20,687 CpG sites into nine modules. Despite the fact that DNA methylation in blood leukocytes is representative of DNA methylation in other tissues [66], it is influenced by cell composition. However, the impact of cell composition was not taken into account in the present analyses. In a previous paper by our group, cell heterogeneity was predicted using DNA methylation levels, and it estimated proportions of six different cell types [24]. These six additional cofounders were not included in the present analysis in order to prevent the reduction of the statistical power to detect significant associations and correlations. Finally, our study did not account for diet, physical activity, smoking, and alcohol consumption of the participants, all of which may affect circulating carotenoid concentrations [67,68].

## 5. Conclusions

In conclusion, DNA methylation levels of 20,687 CpG sites were associated with plasma total carotenoid concentrations. Using WGCNA, nine co-methylation modules were identified. A total of 2734 hub genes, and more specifically 17 unique top hub genes, associated with total carotenoid concentrations were potentially related to lipid profile traits. Even though further studies and validation of the hub genes are needed, this research provides evidence for the potential role of gene co-methylation in the relationship between carotenoids, lipid profile and ultimately CM health.

## Figures and Tables

**Table 1:** Characteristic and biochemical parameters.

Biochemical Parameters	Parents (*n* = 22)	Children (*n* = 26)
Age (years)	42.3 ± 5.3	11.3 ± 3.4
BMI (kg/m^2^)	23.9 ± 3.0	-
BMI percentile	-	50 ± 31.1
TC (mmol/L)	4.68 ± 0.55	4.28 ± 0.51
HDL-C (mmol/L)	1.63 ± 0.38	1.55 ± 0.29
LDL-C (mmol/L)	2.61 ± 0.55	2.31 ± 0.45
TC/HDL-C	3.02 ± 0.78	2.83 ± 0.47
ApoB100 (g/L)	0.80 ± 0.15	0.70 ± 0.13
TG (mmol/L)	0.95 ± 0.35	0.93 ± 0.39
Glucose (mmol/L)	5.20 ± 0.34	4.86 ± 0.23
Insulin (pmol/L)	66.59 ± 33.54	73.00 ± 28.96
CRP (mg/L)	0.77 ± 0.84	0.43 ± 0.66
Total carotenoids (μmol/L)	6.35 ± 2.39	5.70 ± 2.05

All values are means ± SD. Abbreviations: Apolipoprotein B100 (ApoB100), body mass index (BMI), C-reactive protein (CRP), high-density lipoprotein cholesterol (HDL-C), low-density lipoprotein cholesterol (LDL-C), standard deviation (SD), total cholesterol (TC), total cholesterol/HDL-C (TC/HDL-C), triglycerides (TG).

**Table 2:** Top hub genes identified from WGCNA analysis.

Module-Lipid	CpG Site	Gene (Chr, Accession Number)	GS	*p*-Value GS	MM	*p*-Value MM
Black HDL-C	cg01999908	*PAX4* (Chr7, NM_006193)	−0.37	0.0092	0.85	2.10 × 10^−14^
	cg08731068	*TBC1D16* (Chr17, NM_019020)	−0.37	0.011	0.89	1.70 × 10^−17^
	cg04010296	*PGA5* (Chr11, NM_014224)	−0.36	0.011	0.83	4.08 × 10^−13^
Turquoise TC	cg00000029	*RBL2* (Chr16, NM_005611)	−0.59	8.49 × 10^−6^	0.83	3.45 × 10^−13^
	cg02500883	*GRIN3A* (Chr9, NM_133445)	−0.57	1.98 × 10^−5^	0.81	2.52 × 10^−12^
	cg00864012	*TEX2* (Chr17, NM_018469)	−0.56	3.39 × 10^−5^	0.85	2.39 × 10^−14^
Turquoise HDL-C	cg27616007	*LST1* (Chr6, NR_029461)	−0.42	0.0027	0.81	3.82 × 10^−12^
	cg24240870	*GOSR1* (Chr17, NM_004871)	−0.42	0.0030	0.81	4.55 × 10^−12^
	cg18089000	*GBGT1* (Chr9, NM_021996)	−0.42	0.0031	0.86	3.88 × 10^−15^
Turquoise LDL-C	cg07665923	*C7orf50* (Chr7, NM_001134396)	−0.45	0.0012	0.83	2.22 × 10^−13^
	cg02119755	*PDPK1* (Chr16, NM_031268)	−0.44	0.0017	0.87	1.62 × 10^−15^
	cg06142108	*SYT17* (Chr16, NM_016524)	−0.43	0.0022	0.88	1.75 × 10^−16^
Turquoise ApoB100	cg17053201	*GABBR1* (Chr6, NM_021904)	−0.46	0.00096	0.82	8.40 × 10^−13^
	cg21671607	*RAG2* (Chr11, NM_000536)	−0.46	0.0011	0.84	7.35 × 10^−14^
	cg07665923	*C7orf50* (Chr7, NM_001134396)	−0.46	0.0011	0.83	2.22 × 10^−13^
Pink HDL-C	cg15505294	*RNASE11* (Chr14, NM_145250)	0.36	0.012	−0.95	1.87 × 10^−24^
	cg01719157	*TRIM68* (Chr11, NM_018073)	−0.35	0.013	0.81	4.10 × 10^−12^
	cg09113768	*DEPDC1* (Chr1, NM_017779)	−0.35	0.015	0.83	2.22 × 10^−13^

Abbreviations: Apolipoprotein B100 (ApoB100), chromosome (Chr), cytosine-phosphate-guanine (CpG), gene significance (GS), high-density lipoprotein cholesterol (HDL-C), low-density lipoprotein cholesterol (LDL-C), module membership (MM), total cholesterol (TC) weighted gene correlation network analysis (WGCNA).

## Supplementary Materials

The following are available online at https://www.mdpi.com/2072-6643/11/6/1265/s1, Figure S1: Scale independence and mean connectivity; Figure S2: Scatterplots of gene significance for lipid profile traits and module membership in the A) black-HDL-C B) pink-HDL-C C) turquoise-TC D) turquoise-HDL-C E) turquoise-LDL-C and F) turquoise-ApoB100; Table S1: Concentrations of plasma carotenoids (µmol/L of plasma); Table S2: CpG sites showing significant correlation with plasma total carotenoids (*n* = 20,687); Table S3: Hub genes identified from WGCNA analysis (*n* = 2734); Table S4: Molecular functions and pathways in the black, turquoise and pink modules.
