# Supplementary material for: Network Analysis of the Potential Role of DNA Methylation in the Relationship between Plasma Carotenoids and Lipid Profile
Source: Nutrients. 2019 Jun 4;11(6):1265. doi: 10.3390/nu11061265 (PMC6628241; doi:10.3390/nu11061265)
Supplement: Supplementary file 1 [file nutrients-11-01265-s001.zip › Supplementary Table 4. Functions and pathwaysdocxv2.docx]

| **Supplementary Table S4. Molecular functions and pathways of genes in the black, turquoise, and pink modules** | | |
| --- | --- | --- |
| Genes | GO molecular functions 2018 | KEGG Pathways 2019 |
| Genes in the black module (n=14) | | |
| ***ANKRD5*** |  |  |
| *FLJ39653* |  |  |
| ***HCFC1R1*** |  |  |
| *LOC729799* |  |  |
| *LTBP4* | transmembrane receptor protein serine/threonine kinase activity (GO:0004675),transforming growth factor beta binding (GO:0050431), integrin binding (GO:0005178), transforming growth factor beta-activated receptor activity (GO:0005024), metal ion binding (GO:0046872), calcium ion binding (GO:0005509) |  |
| *MAD1L1* |  | Cell cycle  Human T-cell leukemia virus 1 infection  Oocyte meiosis  Progesterone-mediated oocyte maturation  Viral carcinogenesis |
| *MAPT* | phosphatidylinositol binding (GO:0035091),SH3 domain binding (GO:0017124), AT DNA binding (GO:0003680), protein phosphatase binding (GO:0019903), sequence-specific DNA binding (GO:0043565), lipoprotein particle binding (GO:0071813), protein phosphatase 2A binding (GO:0051721), Hsp90 protein binding (GO:0051879),  tubulin binding (GO:0015631), protein kinase binding (GO:0019901), phosphatidylinositol bisphosphate binding (GO:1902936), microtubule binding (GO:0008017), double-stranded DNA binding (GO:0003690), sequence-specific double-stranded DNA binding (GO:1990837), RNA binding (GO:0003723), DNA binding (GO:0003677).  actin binding (GO:0003779), dynactin binding (GO:0034452), protein homodimerization activity (GO:0042803), single-stranded DNA binding (GO:0003697), kinase binding (GO:0019900), phosphatidylinositol phosphate binding (GO:1901981) | Alzheimer disease  MAPK signaling pathway |
| ***MUC2*** |  | Amoebiasis  Gastric cancer |
| ***NAP1L4*** | RNA binding (GO:0003723) |  |
| *PGA5* | endopeptidase activity (GO:0004175), aspartic-type peptidase activity (GO:0070001), aspartic-type endopeptidase activity (GO:0004190) | Protein digestion and absorption |
| *SEMA5B* | neuropilin binding (GO:0038191) | Axon guidance |
| ***SGCD*** |  | Arrhythmogenic right ventricular cardiomyopathy (ARVC)  Dilated cardiomyopathy (DCM)  Hypertrophic cardiomyopathy (HCM)  Viral myocarditis |
| *TCIRG1* | ATPase binding (GO:0051117), hydrogen-exporting ATPase activity (GO:0036442), hydrogen ion transmembrane transporter activity (GO:0015078), ATPase activity, coupled to transmembrane movement of ions, rotational mechanism (GO:0044769), proton-transporting ATPase activity, rotational mechanism (GO:0046961) | Collecting duct acid secretion  Epithelial cell signaling in Helicobacter pylori infection  Human papillomavirus infection  Lysosome  Oxidative phosphorylation  Phagosome  Rheumatoid arthritis  Synaptic vesicle cycle  Tuberculosis  Vibrio cholerae infection |
| *TSSC1* |  |  |
| Genes in the turquoise module (n=20) | | |
| *ACAD8* | acyl-CoA dehydrogenase activity (GO:0003995) | Valine, leucine and isoleucine degradation |
| *AGXT* | amino acid binding (GO:0016597), carboxylic acid binding (GO:0031406), protein homodimerization activity (GO:0042803), transaminase activity (GO:0008483) | Alanine, aspartate and glutamate metabolism  Glycine, serine and threonine metabolism  Glyoxylate and dicarboxylate metabolism  Peroxisome |
| *ANKRD33B* |  |  |
| *CDH23* |  |  |
| *FOLR3* |  | Endocytosis |
| *GALNS* | sulfuric ester hydrolase activity (GO:0008484) | Glycosaminoglycan degradation  Lysosome |
| *HIP1* | phosphatidylinositol binding (GO:0035091), phosphatidylinositol-3-phosphate binding (GO:0032266), clathrin adaptor activity (GO:0035615), phosphatidylinositol bisphosphate binding (GO:1902936), glutamate receptor binding (GO:0035254), AP-2 adaptor complex binding (GO:0035612), actin binding (GO:0003779), protein homodimerization activity (GO:0042803), phosphatidylinositol-3,4-bisphosphate binding (GO:0043325), actin filament binding (GO:0051015).  phosphatidylinositol-3,5-bisphosphate binding (GO:0080025), protein heterodimerization activity (GO:0046982), epidermal growth factor receptor binding (GO:0005154), clathrin light chain binding (GO:0032051), endocytic adaptor activity (GO:0098748), growth factor receptor binding (GO:0070851), phosphatidylinositol phosphate binding (GO:1901981) | Huntington disease |
| *KY* |  |  |
| ***LGALS12*** |  |  |
| *LMO2* | transcription regulatory region DNA binding (GO:0044212), transcriptional activator activity, RNA polymerase II transcription regulatory region sequence-specific binding (GO:0001228), bHLH transcription factor binding (GO:0043425), transcription regulatory region sequence-specific DNA binding (GO:0000976), RNA polymerase II transcription factor binding (GO:0001085), transcriptional repressor activity, RNA polymerase II activating transcription factor binding (GO:0098811), RNA polymerase II regulatory region DNA binding (GO:0001012), activating transcription factor binding (GO:0033613), E-box binding (GO:0070888), transcriptional activator activity, RNA polymerase II transcription factor binding (GO:0001190), RNA polymerase II activating transcription factor binding (GO:0001102), RNA polymerase II regulatory region sequence-specific DNA binding (GO:0000977) | Transcriptional misregulation in cancer |
| *LPAR6* | G-protein coupled receptor activity (GO:0004930), bioactive lipid receptor activity (GO:0045125) | Neuroactive ligand-receptor interaction  PI3K-Akt signaling pathway  Pathways in cancer  Phospholipase D signaling |
| *MAPT* | phosphatidylinositol binding (GO:0035091), SH3 domain binding (GO:0017124), AT DNA binding (GO:0003680), protein phosphatase binding (GO:0019903), sequence-specific DNA binding (GO:0043565), lipoprotein particle binding (GO:0071813), protein phosphatase 2A binding (GO:0051721), Hsp90 protein binding (GO:0051879), tubulin binding (GO:0015631), protein kinase binding (GO:0019901), phosphatidylinositol bisphosphate binding (GO:1902936), microtubule binding (GO:0008017), double-stranded DNA binding (GO:0003690). sequence-specific double-stranded DNA binding (GO:1990837), RNA binding (GO:0003723), DNA binding (GO:0003677), actin binding (GO:0003779), dynactin binding (GO:0034452), protein homodimerization activity (GO:0042803), single-stranded DNA binding (GO:0003697), kinase binding (GO:0019900), phosphatidylinositol phosphate binding (GO:1901981) | Alzheimer disease  MAPK signaling |
| *MARCH8* | ubiquitin-protein transferase activity (GO:0004842) |  |
| *NDUFS2* | NADH dehydrogenase (ubiquinone) activity (GO:0008137), NADH dehydrogenase (quinone) activity (GO:0050136), ubiquitin protein ligase binding (GO:0031625), NADH dehydrogenase activity (GO:0003954), ubiquitin-like protein ligase binding (GO:0044389) | Alzheimer disease  Huntington disease  Non-alcoholic fatty liver disease (NAFLD)  Oxidative phosphorylation  Parkinson disease  Retrograde endocannabinoid signaling  Thermogenesis |
| ***PACS2*** |  |  |
| *PFDN6* |  |  |
| *PVRL4* |  |  |
| *SREBF1* | transcription regulatory region DNA binding (GO:0044212), DNA binding (GO:0003677), transcription regulatory region sequence-specific DNA binding (GO:0000976), RNA polymerase II regulatory region DNA binding (GO:0001012), RNA polymerase II regulatory region sequence-specific DNA binding (GO:0000977). | AMPK signaling pathway  Insulin resistance  Insulin signaling pathway  Non-alcoholic fatty liver disease (NAFLD) |
| *UACA* |  |  |
| *YIF1A* |  |  |
| Genes in the pink module (n=13) | | |
| ***CNNM2*** |  |  |
| *SNTB1* |  |  |
| *CLEC16A* | Rab GTPase binding (GO:0017137), Ras GTPase binding (GO:0017016) |  |
| *GABBR1* | G-protein coupled receptor activity (GO:0004930), GABA receptor activity (GO:0016917) | Estrogen signaling pathway  GABAergic synapse  Morphine addiction  Neuroactive ligand-receptor interaction  Taste transduction  cAMP signaling |
| ***FAM188A*** |  |  |
| *LRIT1* |  |  |
| *PTDSS2* |  | Glycerophospholipid metabolism |
| *AQP12B* | water transmembrane transporter activity (GO:0005372), substrate-specific channel activity (GO:0022838), water channel activity (GO:0015250) |  |
| *FABP12* |  |  |
| *GAB2* | phosphatidylinositol bisphosphate binding (GO:1902936), phosphatidylinositol-3,4,5-trisphosphate binding (GO:0005547), phosphatidylinositol-3,4-bisphosphate binding (GO:0043325), phosphatidylinositol phosphate binding (GO:1901981) | Chronic myeloid leukemia  Fc epsilon RI signaling pathway  Fc gamma R-mediated phagocytosis  Osteoclast differentiation  Phospholipase D signaling pathway  Ras signaling pathway  Sphingolipid signaling |
| *KIF6* | motor activity (GO:0003774), tubulin binding (GO:0015631), microtubule binding (GO:0008017), nucleoside-triphosphatase activity (GO:0017111), ATPase activity (GO:0016887), microtubule motor activity (GO:0003777) |  |
| *MGAM* |  | Carbohydrate digestion and absorption  Galactose metabolism  Starch and sucrose metabolism |
| *ZNF561* |  | Herpes simplex virus 1 infection |
| Analyses of GO molecular functions and KEGG pathways were conducted using Enrichr. Central genes are in bold. | | |
