# Supplementary figures and images for: Network Analysis of the Potential Role of DNA Methylation in the Relationship between Plasma Carotenoids and Lipid Profile
Source: Nutrients. 2019 Jun 4;11(6):1265. doi: 10.3390/nu11061265 (PMC6628241; doi:10.3390/nu11061265)

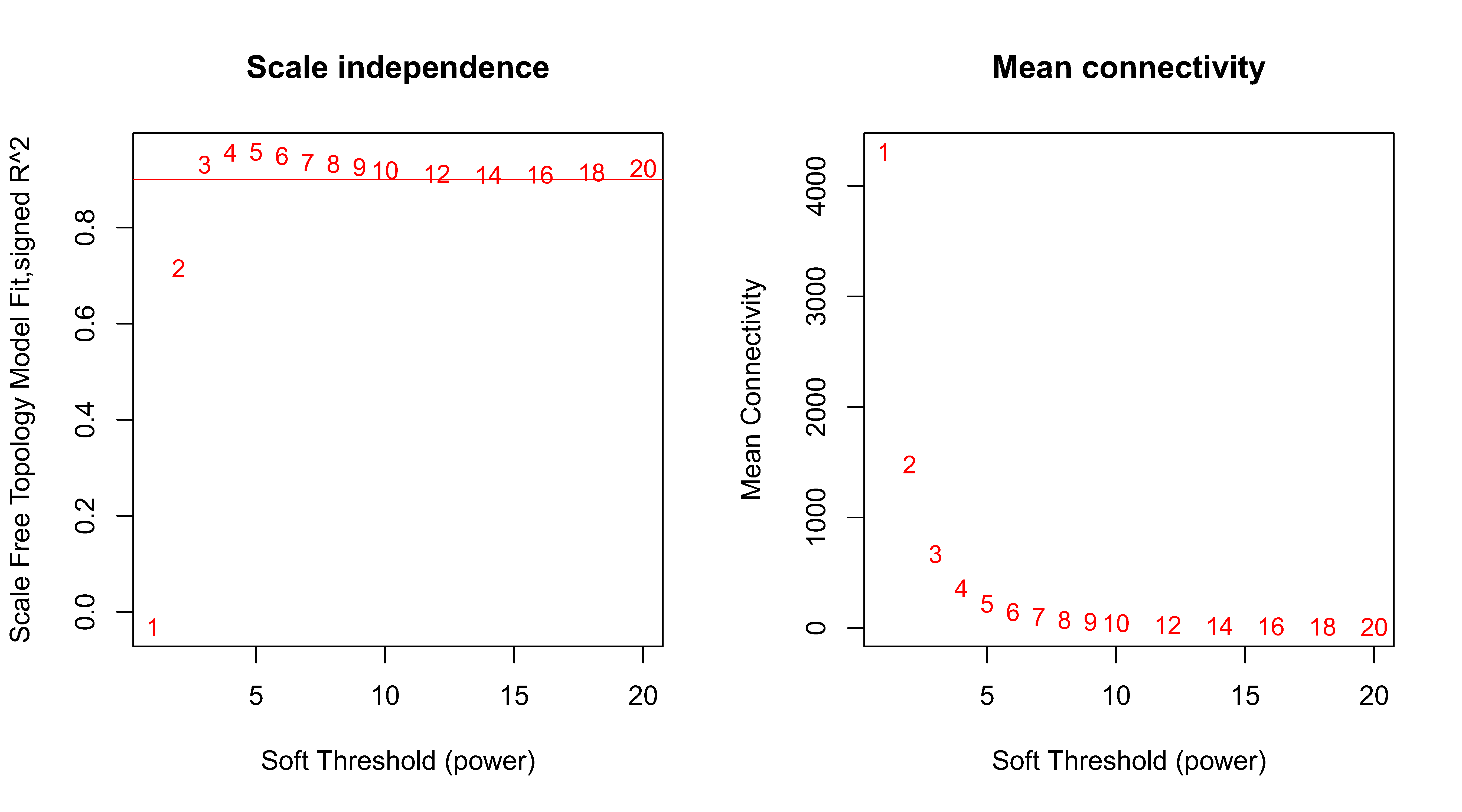

Supplement: Supplementary file 1 [file nutrients-11-01265-s001.zip › Supplementary Figure 1_Nutrientsv2.tiff]

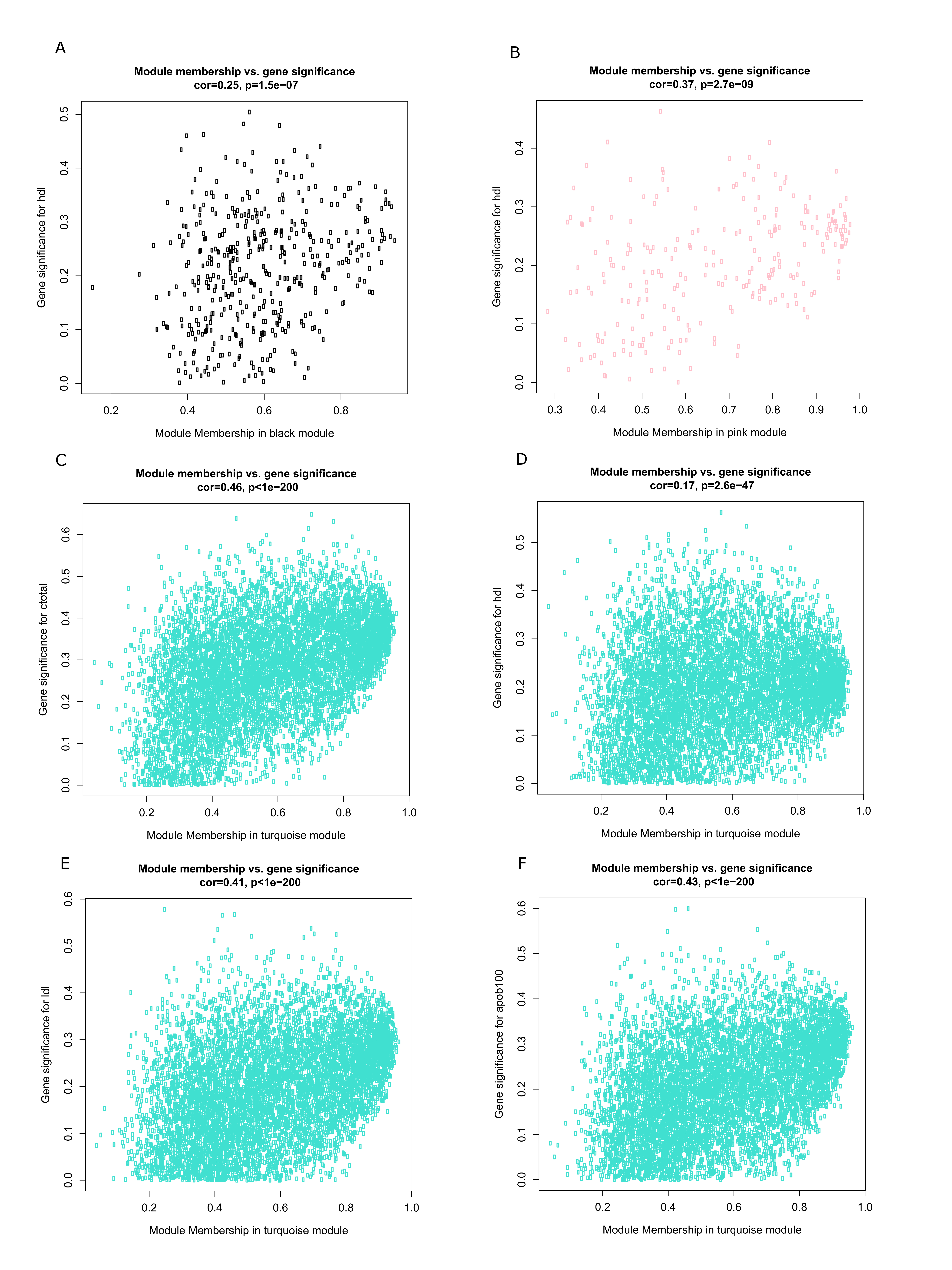

Supplement: Supplementary file 1 [file nutrients-11-01265-s001.zip › Supplementary figure 2_Nutrientsv2.tiff]
